# IgG4-related hypertrophic pachymeningitis with tumor-like intracranial and intracerebral lesions

**DOI:** 10.1007/s00701-022-05340-5

**Published:** 2022-08-17

**Authors:** Majid Esmaeilzadeh, Mete Dadak, Oday Atallah, Nora Möhn, Thomas Skripuletz, Christian Hartmann, Rozbeh Banan, Joachim K. Krauss

**Affiliations:** 1 Department of Neurosurgery, Hannover Medical School, Carl-Neuberg-Street 1, 30625 Hannover, Germany; 2 Institute for Diagnostic and Interventional Neuroradiology, Hannover Medical School, Hannover, Germany; 3 Clinic of Diagnostic and Interventional Radiology and Neuroradiology, St. Vincenz-Hospital, Paderborn, Germany; 4 Department of Neurology, Hannover Medical School, Hannover, Germany; 5 Institute of Neuropathology, Hannover Medical School, Hannover, Germany

**Keywords:** Pachymeningitis, IgG4-related disease, Inflammation, Central nervous system, Surgery

## Abstract

**Objective:**

IgG4-related hypertrophic pachymeningitis is a rare fibroinflammatory disorder that may cause localized or diffused thickening of the dura mater. Misinterpretations of the clinical and imaging findings are common. Clinical manifestations depend on the location of the inflammatory lesion and on compression of neural structures leading to functional deficits. A dural biopsy is commonly needed for a definitive diagnosis. Immunomodulatory therapy is considered the therapy of choice.

**Methods:**

Four patients with IgG4-related hypertrophic pachymeningitis were identified over a 5-year period. Patient-related characteristics including age, preoperative workup, signs and symptoms of patients, and diagnostic procedures were evaluated. Furthermore, the surgical treatment and 5-year follow-up outcomes were analyzed.

**Results:**

There were two adults and two adolescents (mean age 32 years; range 15 to 67 years). Two patients were male, and two were female. No history of disease was known in any of the patients. Clinical symptoms were epilepsy (*n* = 2), ataxia and nausea (*n* = 1), and facial nerve palsy (*n* = 1). MR imaging studies showed contrast enhancing lesions in the temporal region in two patients, and in the cerebellar region in the other two patients. Subtotal resection was performed in two instances and a biopsy via a suboccipital retrosigmoid approach was obtained in the other two patients. Histochemical and immunohistochemical investigations revealed an IgG 4 disease in all of these patients. Immunomodulatorry therapy led to clinical stability during follow-up of 5 years in all four cases.

**Conclusion:**

The diagnosis of IgG4-related hypertrophic pachymeningitis is challenging, but is of great relevance as treatment differs significantly from other forms of pachymeningitis and a specific therapeutic approach may avoid long-term neurological complications. Our series contributes to a better clinical characterization of this rare disease.

## Introduction

Immunoglobulin G (IgG)-4-related disease is an uncommon chronic inflammatory disorder, which may affect various organs. It is characterized by distinct morphological and histological features with dense lymphoplasmocytic IgG 4-positive infiltrates [18, 23]. Over the last decade, IgG4-related disease was recognized to occur in various organs including pancreas, lung, thyroid, lymph nodes, retroperitoneum, aorta, and as well the central nervous systems (CNS) [21]. Hypophysitis and hypertrophic pachymeningitis present the most common CNS manifestations of this rare disease [2].

IgG4-related hypertrophic pachymeningitis accounted for 8.8% out of 159 cases in a study on hypertrophic pachymeningitis in Japan [35]. It manifests as localized or diffused thickening of the dura mater [23, 27]. Clinical manifestations depend on the location of the inflammatory lesion and on compression of neural structures leading to functional deficits [3, 5]. A dural biopsy is commonly needed to establish a definite diagnosis [2, 30]. Immunomedulatory treatment is considered the treatment of choice and was suggested to avoid disease recurrence [7, 24, 27].

Early diagnosis and treatment are pivotal to prevent persistent neurological damage but misinterpretations of the clinical and imaging findings are frequent [22]. Rarely, IgG4-related pachymeningitis presents with atypical MRI findings including tumor-like intracranial masses mimicking meningioma [16, 29]. In such cases, the diagnosis of hypertrophic pachymeningitis is challenging.

Here, we outline the clinical and imaging characteristics, the therapeutic approaches, and long-term outcomes of four patients with IgG4-related hypertrophic pachymeningitis with tumor-like intracranial and intracerebral lesions.

## Materials and methods

To identify patients with a diagnosis of IgG4-related intracranial disease, a systematic review of the databank of the Department of Neurosurgery and the Institute of Neuropathology at Hannover Medical School was performed over a 5-year period.

Four patients were identified, and the charts with demographic and clinical data (age, sex, preoperative signs and symptoms, concomitant diseases), histopathological findings, and preoperative and postoperative imaging studies were evaluated. In none of the patients, IgG4-related disease was known previously, and the primary goal of surgery was to obtain a histopathological diagnosis. Neurosurgical procedures were performed using departmental standard techniques as described elsewhere [9–11]. Surgical results and outcomes were analyzed, and all patients were followed-up for up to 5 years after hospital discharge. Patient outcome was assessed by the Karnofsky performance status (KPS) score.

Histological diagnosis of IgG4-related pachymeningitis was based on the consensus criteria described by Deshpande et al. [6].

## Results

The demographical and clinical data of the four patients are summarized in Table 1. Two of them were adults and two were adolescents. The mean age at presentation was 32 years (range, 15 to 67 years). Two patients were male, and two were female. Clinical symptoms leading to diagnosis were epilepsy (*n* = 2), ataxia and nausea (*n* = 1), and facial nerve palsy (*n* = 1). One patient had a history of p-ANCA-positive polyangiitis, while there were no known inflammatory or immunological diseases in the other three patients.

**Table 1:** Summary of demographic and clinical data of 4 patients with intracranial IgG4-related hypertrophic pachymeningitis and tumor-like lesions

Patient Nr	Age (years)	Sex	Symptoms at presentation	Serum IgG4 concentration (0.08–1.4 g/l)	Organ system involvement
1	67	F	Facial nerve palsy	4.71	None
2	30	M	Epilepsy	0.77	None
3	16	F	Ataxia and nausea	0.27	None
4	15	M	Epilepsy	1.11	None

**Fig. 1:**
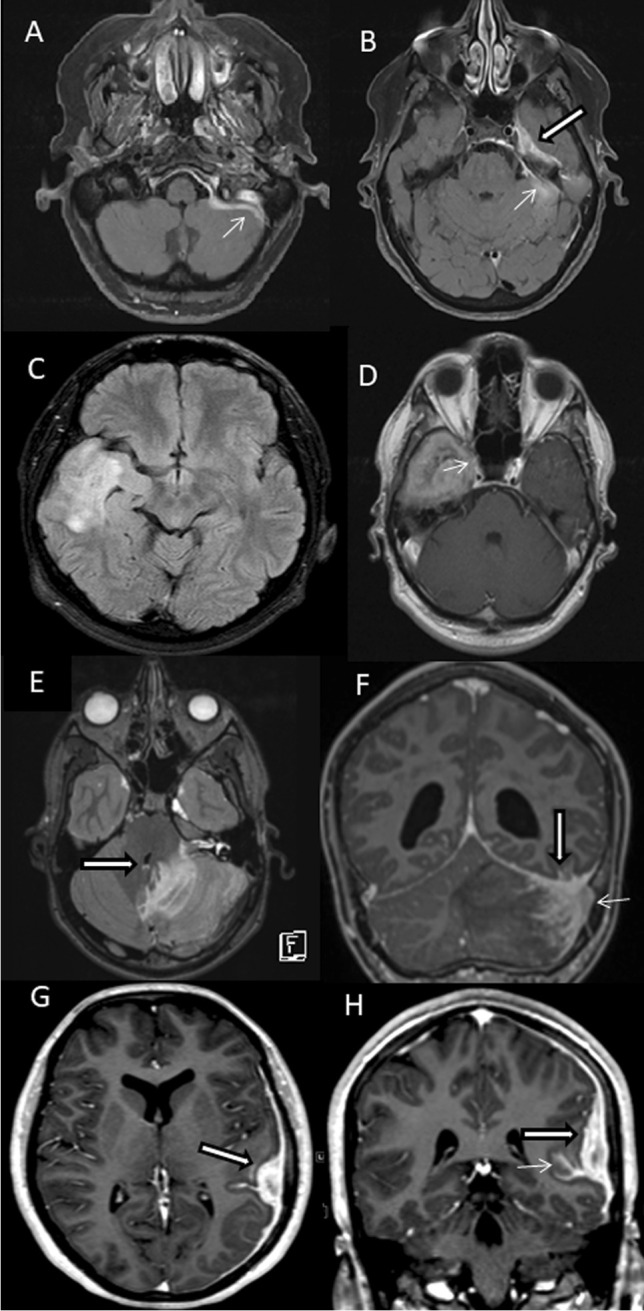
**A**, **B** Patient 1, 67-year-old woman: axial gadolinium (Gd)-enhanced fat-saturated T1-weighted MRI shows a heterogeneously contrast-enhancing extraaxial mass in the left middle fossa (large arrow) with infiltration of the dura extending to the posterior fossa (small arrows). **C**, **D** Patient 2, 30-year-old man: axial FLAIR-weighted MRI shows extensive cerebral edema in the middle and superior temporal gyrus. Axial Gd-enhanced T1-weighted MRI demonstrates homogenous contrast enhancing solid mass in the inferior temporal gyrus extending to the cavernous sinus (small arrow). **E**, **F** Patient 3, 16-year-old woman: T2-weighted axial images show extensive cerebral edema in the left cerebellar hemisphere with compression of the fourth ventricle (large arrow in **E**), and Gd-enhanced fat-saturated T1-weighted MRI shows heterogeneously contrast enhancing process infiltrating the cerebellar sulci, dural sinuses (small arrow), the left sided tentorium cerebelli and the ipsilateral sulci of the inferior temporal gyrus (large arrow in **F**). **G**, **H** Patient 4, 15-year-old man: T1-weighted gadolinium-enhanced axial and coronal MRI show a strongly contrast-enhancing left temporal tumor (large arrows) with finger-like extension to the adjacent sulci (small arrow)

MR imaging showed tumor-like lesions in all four patients with involvement of the adjacent cerebral parenchyma in three of them. Patient 1 had a contrast-enhancing irregularly shaped extraaxial mass in the left middle fossa with infiltration of the dura extending to the posterior fossa (Fig. 1A, B). In patient 2, FLAIR-weighted imaging showed extensive cerebral edema in the right middle and superior temporal gyrus (Fig. 1C) while gadolinium-enhanced T1-weighted imaging revealed a contrast-enhancing solid mass in the inferior temporal gyrus extending to the cavernous sinus (Fig. 1D). In patient 3, T2-weighted images demonstrated extensive cerebral edema in the left cerebellar hemisphere with compression of the fourth ventricle (Fig. 1E) and T1-weighted gadolinium-enhanced imaging showed a heterogeneously contrast-enhancing tumor adjacent to the dura of the transverse sinus (Fig. 1F). In patient 4, T1-weighted gadolinium-enhanced images showed focal thickening of the dura over the left hemisphere and a strongly contrast-enhancing subdural mass lesion with finger-like extension into the adjacent sulci (Fig. 1G, H).

**Fig. 2:**
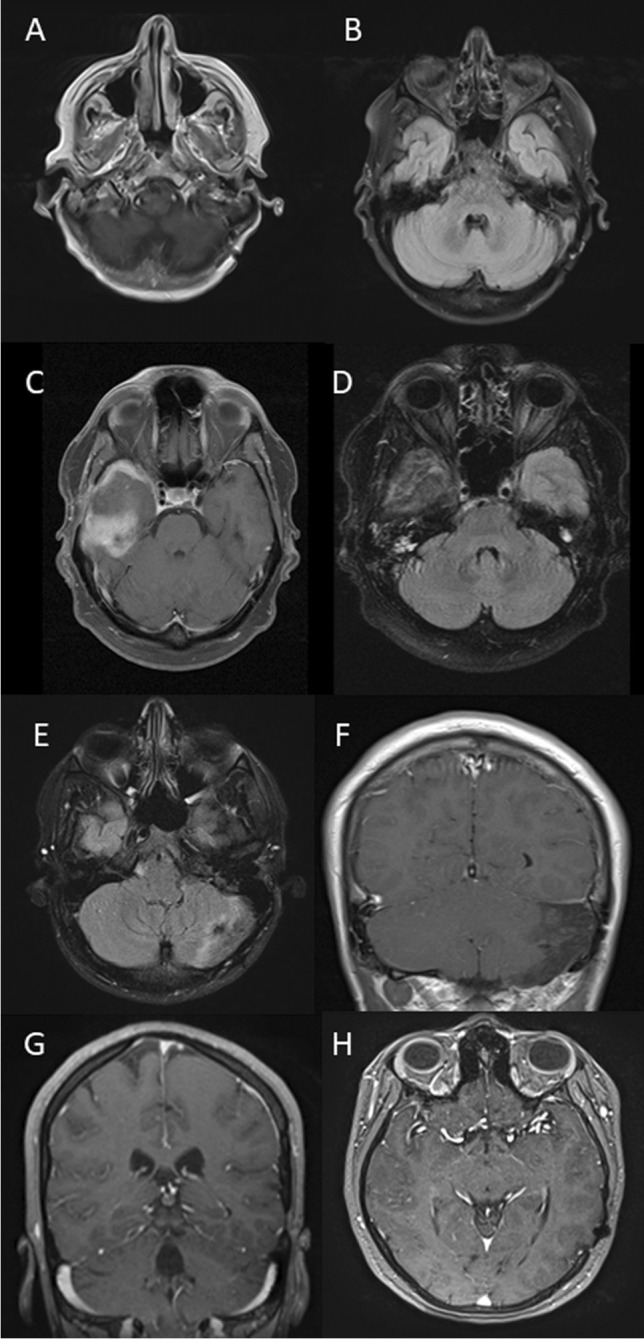
**A**, **B** Patient 1: axial gadolinium (Gd)-enhanced fat-saturated T1-weighted MRI shows a reduction of the mass lesions and of edema 4 years postoperatively. **C**, **D** Patient 2: axial FLAIR-weighted MRI shows significant reduction of cerebral edema in the middle and superior temporal gyrus. Axial Gd-enhanced T1-weighted MRI demonstrates also reduction of the solid mass in the inferior temporal gyrus 2 years postoperatively. **E**, **F** Patient 3: T2-weighted axial images show reduction of cerebral edema in the left cerebellar hemisphere. Gd-enhanced fat-saturated T1-weighted MRI further shows considerable reduction of contrast enhancement 4 years postoperatively. **G**, **H** Patient 4: T1-weighted gadolinium-enhanced axial and coronal MRI do not show a contrast-enhancing tumor any longer 5 year postoperatively

Subtotal resection of the mass lesions was achieved in two patients via osteoplastic craniotomy, and biopsies were obtained via a suboccipital retrosigmoid approach in the other two patients. There were no intraoperative complications. Postoperatively, no new neurological deficits occurred (Fig. 2).

**Fig. 3:**
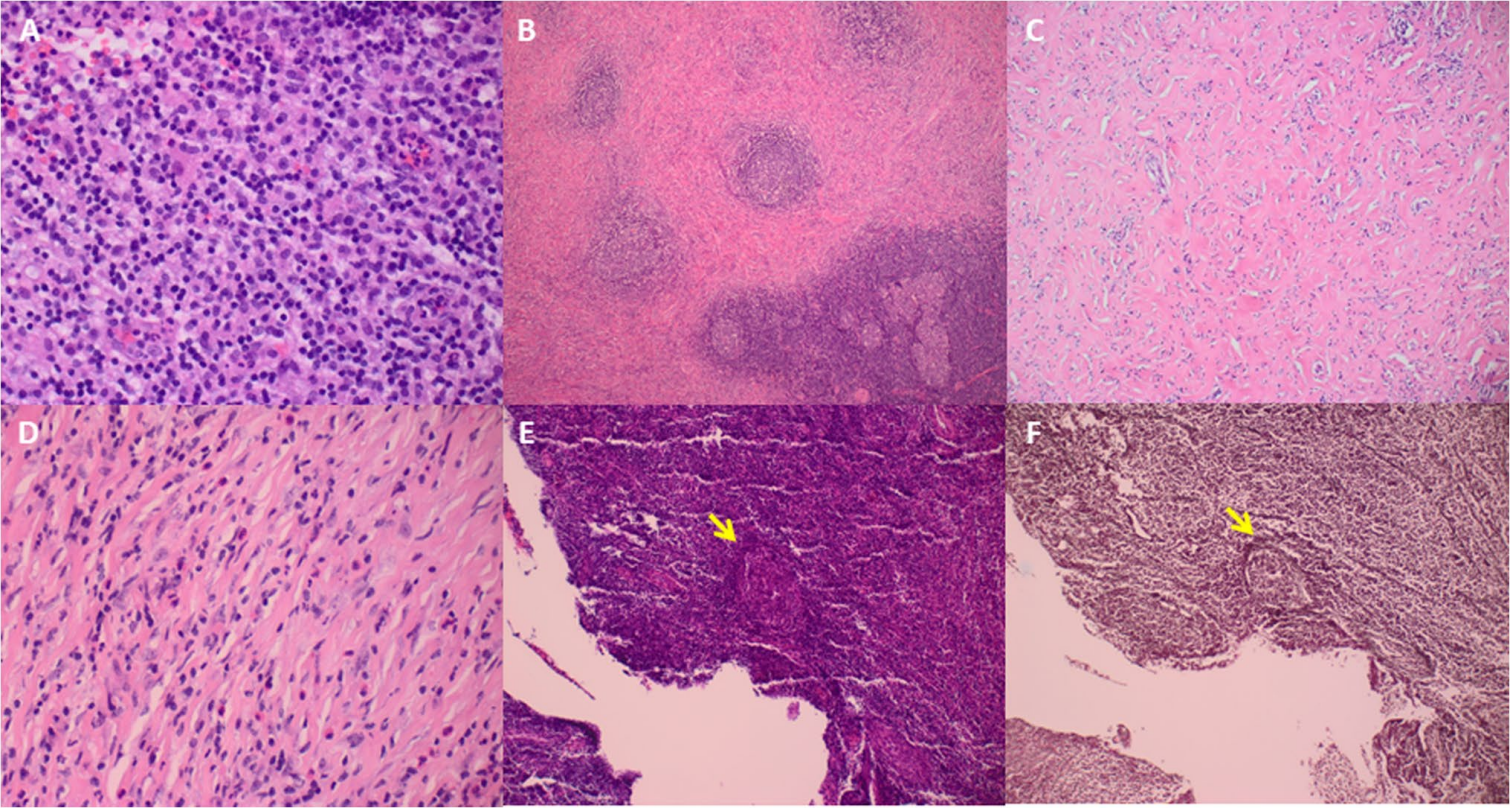
**A** Photomicrographs of sections stained with H&E. Infiltration of inflammatory cells predominantly composed of monomorphic lymphocytes and mature plasma cells (patient 1). **B**, **C** High power views showing patchy infiltrates of lymphoid cells building reactive follicles with germinal center formation. Irregularly whirl-shaped fibrosis (storiform fibrosis) typical for IgG4-related diseases (patient 2). **D** Mild to moderate eosinophilic infiltration is evident (patient 4). **E** Both transmural and luminal aggregation of inflammatory cells in a small vein leading to obliterative phlebitis is present (arrow) (patient 4). **F** Same finding in silver staining

**Fig. 4:**
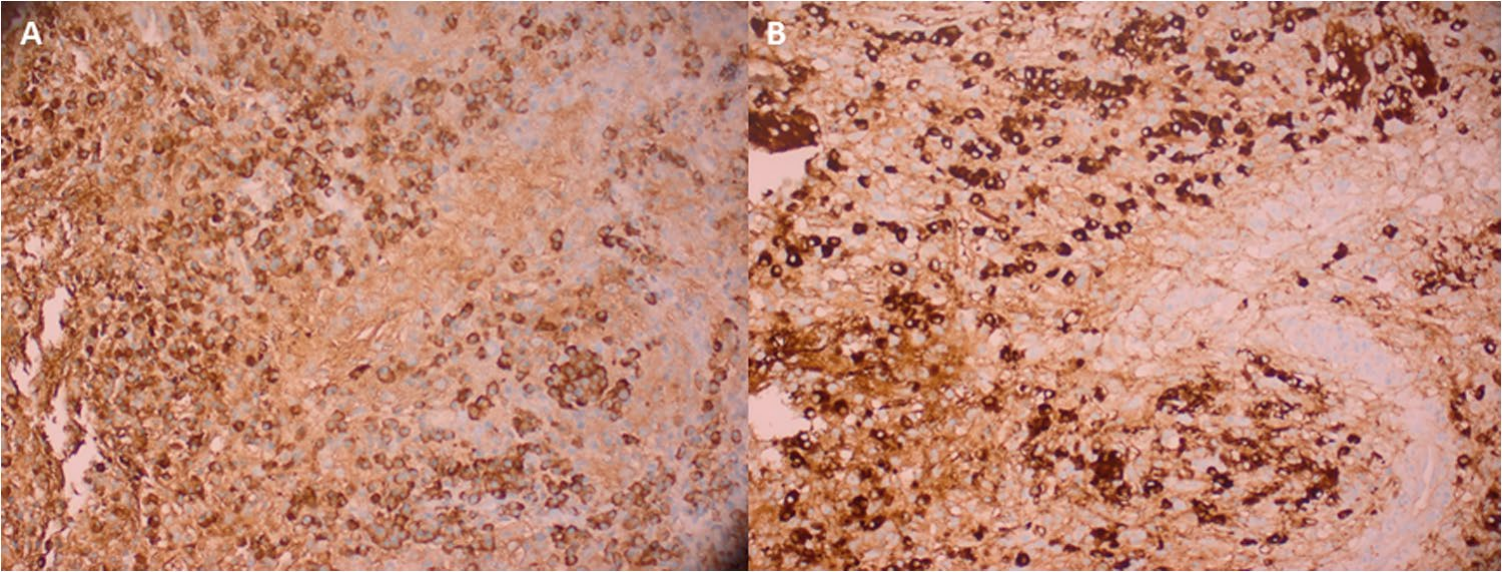
Photomicrographs of sections prepared with immunohistochemical staining for IgG (**A**) and IgG4 (**B**) showing a high number of plasma cells labeled for IgG. Among these abundant (> 100) cells are marked with IgG4-antibodies constituting > 90% of all IgG + plasma cells (patient 3)

Histopathological examination revealed findings of IgG4-related hypertrophic pachymeningitis in all instances. Hematoxylin and Eosin staining showed infiltration of inflammatory cells predominantly composed of monomorphic lymphocytes (Fig. 3A) and mature plasma cells with patchy infiltrates of lymphoid cells building reactive follicles with germinal centers (Fig. 3B). Furthermore, irregularly whirl-shaped fibrosis (Fig. 3C) and mild to moderate eosinophilic infiltration (Fig. 3D) were evident. In addition, both transmural and luminal aggregation of inflammatory cells in small veins resulting in obliterative phlebitis was detected (Fig. 3E, F). A high fraction of plasma cells labeled positively for IgG in all instances. Among these, 90% were stained with IgG4-antibodies (Fig. 4A, B).

Preoperative serum IgG4 levels were not available. The postoperative serum IgG4 level was markedly increased in one patient (case 1) 4.71 g/L (normal range 0.08–1.4 g/L), while this was not the case in the other three patients (Table 2). Investigation for other systemic manifestations of IgG4 related-disease was negative in all patients. The findings and treatment plans were discussed in the interdisciplinary neurooncology board.

**Table 2:** Treatment and outcome of 4 patients with intracranial IgG4-related hypertrophic pachymeningitis and tumor like-lesions

Patient Nr	Neurosurgical treatment	Adjuvant therapy	Outcome at 5-year follow-up
1	Biopsy via suboccipital retrosigmoid craniotomy	Prednisolone 15 mg daily, afterwards reduction of dosage Rituximab 375 mg/m^2^	Facial nerve palsy (KPS score 90%)
2	Subtotal resection via craniotomy	Prednisolone (80 mg daily) for 6 weeks, afterwards reduction of dosage weekly	Asymptomatic (KPS score 100%)
3	Biopsy via suboccipital retrosigmoid craniotomy	Prednisolone 20 mg daily for 3 months, afterwards reduction of dosage weekly Methotrexate 25 mg weekly for 4 years	Asymptomatic (KPS score 100%)
4	Subtotal resection via craniotomy	Prednisolone 30 mg daily for 3 months afterwards reduction of dosage weekly Methotrexate 20 mg weekly for 4 years, afterwards rituximab 1 gr every 6 months Methotrexate 1o mg weekly	Asymptomatic (KPS score 100%)

Postoperative treatment consisted in the administration of glucocorticoids in all patients and of immunosuppressive agents in 3 instances (Table 2). The further clinical course was unremarkable. Follow-up MRI obtained after surgery showed reduction of the mass lesions and of edema (Fig. 2). There was no recurrence up to 5 years after diagnosis in all patients. At the last follow-up, three patients were doing well without any symptoms (KPS score: 100%), and one was mildly disabled (KPS score: 90%).

## Discussion

Our present study demonstrates that IgG4-related hypertrophic pachymeningitis may not only manifest as tumor-like intracranial mass lesions but it may also affect adjacent brain tissue. MRI findings in our patients were misleading and did not show the typical features of IgG 4-related hypertrophic pachymeningitis. Remarkably, in all instances, there were no other previous clinical manifestations of IgG4-related disease. Only few cases with tumor-like intracranial lesions as a manifestation of IgG4-related hypertrophic pachymeningitis have been published previously [7, 13, 15–17, 25, 26]. It is important to obtain a definitive histopathological diagnosis in such patients in order to avoid delayed and unnecessary treatment.

The full recognition of IgG4-related disease was only achieved in 2001 in patients with sclerosing pancreatitis and elevated serum IgG4 concentrations [8]. After IgG4-related disease manifestations were found in several other organs, it became apparent that the immunopathological pathomechanisms are also present in some patients with hypertrophic pachymeningitis and hypophysitis, which both had been considered “idiopathic” before [20].

Overall, IgG4-related hypertrophic pachymeningitis is a rare disorder that has been diagnosed more frequently in Japan [12, 14]. Differential diagnoses, in particular in cases with tumor-like intracranial manifestations, include lymphoma, sarcoidosis, and other immunological or infectious granulomatous diseases [3, 20]. From a clinical point of view, the diagnosis should be based on radiological and laboratory findings as well as on the effectiveness of corticosteroid or immunosuppressive therapy [2, 12]. Meningeal biopsy, however, is considered to provide the determinant diagnostic yield in this scenario [1, 2].

According to an international multidisciplinary consortium, a definitive histopathological diagnosis of IgG4-related disease requires the presence of two out of three morphological histological criteria: lympho-plasmacellular infiltrates with IgG4-positive plasma cells, storiform fibrosis, and obliterative phlebitis [6]. While the storiform remodeling of the collagenous fiber structures of the dura mater rarely presents a diagnostic challenge from a histopathological point of view, it may be difficult to detect obliterative phlebitis or sufficient IgG4-positive plasma cells in small biopsies. With regard to the latter criterion, either an IgG4:IgG ratio greater than 40% or the detection of more than 10 IgG4-positive cells in a high power field has been recommended for confirmation of the diagnosis [19]. In addition, if only one of the histopathological criteria for the diagnosis of IgG4-related hypertrophic pachymeningitis is present, it has been proposed to consider also serum IgG4 concentrations and the possible involvement of other organs [20]. It also needs to be mentioned that the differentiation of IgG4-related hypertrophic pachymeningitis from a lympho-plasmocyte-rich meningioma WHO grade I can be challenging, especially using frozen sections, when the specimens were obtained under the assumption of resecting such a tumor [6, 20].

The most common neurological symptoms of patients suffering from IgG4-related hypertrophic pachymeningitis are headache and cranial nerves palsies, followed by motor or sensory deficits, seizures, and ataxia. Headache may reflect meningeal inflammation [2, 34]. Cranial nerve palsies are usually due to compression by the thickened dura mater [4, 23]. Clinical signs and symptoms, however, do not reflect only compression by meningeal thickening but also secondary vascular damage caused by vessel compression [4, 23].

MRI has been considered the best option for diagnostic purposes and treatment monitoring. The common radiological feature of hypertrophic pachymeningitis is linear dural thickening often associated with extension to the neighboring leptomeninges. While cranial nerves may be involved, pachymeningeal bulging masses mimicking tumors are less common [21, 32]. Gadolinium-enhanced T1-weighted MR images allow the identification of active inflammation along the cerebral meninges [21] and the rare involvement of brain parenchyma. FLAIR- and T2-weighted imaging reveals the amount of cerebral edema. A rare complication shown by MRI is thrombosis of dural sinuses [28, 33]. Computed tomography is a helpful imaging modality in the assessment of cranial nerve involvement in the skull base [1, 20].

There are no standard guidelines for the treatment of IgG4-related hypertrophic pachmeningitis. Wallace et al. recommended glucocorticoid therapy after confirmation of the diagnosis, followed by the addition of immunosuppressive agents in the event of recurrence [31]. High-dose methylprednisolone therapy (1 g/day for 3 days) has been recommended in patients with acute severe neurological deficits [2]. Another strategy is the concomitant use of immunosuppressive agents such as methotrexate (20 mg/week), azathioprine (100–200 mg/day), mycophenolate mofetil (1000 mg twice daily), and cyclophosphamide (either oral 100 mg/day) [21, 34]. In our series, adjuvant therapy was used as a steroid-sparing strategy in all instances.

The present study has some limitations. First, the data which was used for this study was evaluated retrospectively. Second, it would have been advantageous to know preoperative IgG4 levels and to obtain longitudinal follow-up of lab examinations.

## Conclusion

There are various patterns of clinical and imaging features in patients with intracranial IgG4-related hypertrophic pachymeningitis which may impede prompt diagnosis. In particular, regarding that neurological signs and symptoms may appear as the first and only clinical manifestations diagnosis is challenging. More experience is needed to develop definitive diagnostic and treatment recommendations for the management of IgG4-related hypertrophic pachymeningitis.

## Abbreviations

IgG: Immunoglobulin G
CNS: Central nervous systems
KPS: Karnofsky performance status
MRI: Magnetic resonance imaging
